# Bim Nuclear Translocation and Inactivation by Viral Interferon Regulatory Factor

**DOI:** 10.1371/journal.ppat.1001031

**Published:** 2010-08-05

**Authors:** Young Bong Choi, John Nicholas

**Affiliations:** Sidney Kimmel Comprehensive Cancer Center, Department of Oncology, Johns Hopkins University School of Medicine, Baltimore, Maryland, United States of America; University of Alberta, Canada

## Abstract

Viral replication efficiency is in large part governed by the ability of viruses to counteract pro-apoptotic signals induced by infection of the host cell. Human herpesvirus 8 (HHV-8) uses several strategies to block the host's innate antiviral defenses via interference with interferon and apoptotic signaling. Contributors include the four viral interferon regulatory factors (vIRFs 1–4), which function in dominant negative fashion to block cellular IRF activities in addition to targeting IRF signaling-induced proteins such as p53 and inhibiting other inducers of apoptosis such as TGFβ receptor-activated Smad transcription factors. Here we identify direct targeting by vIRF-1 of BH3-only pro-apoptotic Bcl-2 family member Bim, a key negative regulator of HHV-8 replication, to effect its inactivation via nuclear translocation. vIRF-1-mediated relocalization of Bim was identified in transfected cells, by both immunofluorescence assay and western analysis of fractionated cell extracts. Also, co-localization of vIRF-1 and Bim was detected in nuclei of lytically infected endothelial cells. *In vitro* co-precipitation assays using purified vIRF-1 and Bim revealed direct interaction between the proteins, and Bim-binding residues of vIRF-1 were mapped by deletion and point mutagenesis. Generation and experimental utilization of Bim-refractory vIRF-1 variants revealed the importance of vIRF-1:Bim interaction, specifically, in pro-replication and anti-apoptotic activity of vIRF-1. Furthermore, blocking of the interaction with cell-permeable peptide corresponding to the Bim-binding region of vIRF-1 confirmed the relevance of vIRF-1:Bim association to vIRF-1 pro-replication activity. To our knowledge, this is the first report of an IRF protein that interacts with a Bcl-2 family member and of nuclear sequestration of Bim or any other member of the family as a means of inactivation. The data presented reveal a novel mechanism utilized by a virus to control replication-induced apoptosis and suggest that inhibitory targeting of vIRF-1:Bim interaction may provide an effective antiviral strategy.

## Author Summary

Human herpesvirus 8 (HHV-8) is a pathogen associated with cancers Kaposi's sarcoma (KS), an endothelial cell disease, and B cell malignancies primary effusion lymphoma and multicentric Castleman's disease. KS is particularly prevalent amongst HIV-positive populations in Africa and is a major health concern. Virus productive replication, in addition to latency, is important for maintaining viral load within the host and also for KS pathogenesis. Essential to HHV-8 and other virus replication is the control of innate host defenses, which comprise stress-sensing cellular signaling pathways that result ultimately in programmed cell death (apoptosis). Here we identify a novel mechanism whereby a viral protein, viral interferon regulatory factor-1 (vIRF-1), mediates inhibition of a stress sensor and initiator of apoptosis, Bim, by inducing its translocation to the cell nucleus and thereby sequestration away from the cytoplasmic compartment where it exerts its pro-death activity. We show that vIRF-1:Bim interaction is necessary for efficient HHV-8 productive replication and that it can be blocked using a cell-permeable antagonist of vIRF-1:Bim binding. Our data not only identify previously unsuspected mechanisms of Bim inactivation and vIRF-1 function, but suggest that inhibitory targeting of vIRF-1 interaction with Bim may be of therapeutic benefit.

## Introduction

Human herpesvirus 8 (HHV-8) is associated with the endothelial tumor Kaposi's sarcoma in addition to the B cell malignancies primary effusion lymphoma (PEL) and multicentric Castleman's disease [1]–[3]. Several genes, including vIRF-1, have been noted to have oncogenic capacity in culture and in *in vivo* models [4], [5]. However, most of these genes are expressed during productive, lytic replication, suggesting that they do not play direct roles in malignant pathogenesis, but rather serve to enhance virus production. Oncogenic properties such as promotion of proliferative signaling pathways and cell survival are indeed consistent with putative roles in establishing conditions that are conducive to virus productive replication. For example, the viral IRFs function to block innate cellular responses of cell cycle arrest and apoptosis that would be induced by virus replication [6]–[8], and these properties, functioning normally to promote virus replication, could also be pro-oncogenic in experimental systems. Of note is that vIRF-1 can bind to and inhibit interferon-activated apoptotic effector proteins such as p53 and GRIM19 [(gene for) retinoid-IFN-induced mortality 19] in addition to p53-activating ATM [9]–[11]. In contrast to investigations of pro-survival and pro-tumorigenic activities of the vIRFs, studies of the functions of these proteins in normal virus biology, and in particular their roles during lytic replication, are lacking, although it is speculated that they do indeed function to enhance virus production by countering innate cellular defenses.

Previous studies from this laboratory noted the importance of the pro-apoptotic BH3-only protein Bim in negative regulation of HHV-8 productive replication in endothelial cells [12]. The viral chemokines vCCL-1 and vCCL-2 were found to induce signal transduction in endothelial cells leading to the repression of Bim induction following starvation-mediated stress and to promote virus replication, effected via both endogenously produced and exogenously added v-chemokines. The central relevance of Bim to productive replication of HHV-8 was indicated more directly by the demonstration that HHV-8 production was massively increased in cells depleted of Bim via shRNA transduction [12]. In this system, the positive effects of vCCL-1 and vCCL-2 were abrogated, suggesting that these viral chemokines exert pro-replication effects via control of lytic cycle-induced Bim expression, thereby acting to inhibit apoptosis and allow a window for virus production.

Bim is induced by a number of stress factors, such as nutrient deprivation, growth factor withdrawal, U.V. irradiation and anti-tumor drugs, in addition to stress induced by virus replication [13]. The classical model of Bim activation is via JNK-mediated phosphorylation, of long (Bim_L_) and extra-long (Bim_EL_) splice-isoforms of Bim, and consequent release from dynein-motor complexes, allowing translocation of the BH3-only protein to mitochondria [14], [15]. Disruption of Bim-cytoskeletal sequestration can also be mediated via induction of Gadd45, regulated by p53 and a mediator of cell cycle arrest and apoptosis [16]; Gadd45 may promote Bim release in part through activation of JNK kinase MEKK4. At mitochondria, Bim triggers apoptosis via interactions with anti-apoptotic Bcl-2 proteins to relieve suppression of Bax/Bak apoptotic effector oligomerization and pore formation in mitochondrial membranes. In addition to cytoplasmic sequestration via dynein motor association, negative regulation of Bim_EL_ can be effected by AKT and ERK phosphorylation, leading to 14-3-3 cytosolic sequestration and proteasomal degradation of Bim, respectively [17]–[19]. The short isoform of Bim, Bim_S_, lacks all phosphorylation sites (JNK, AKT and ERK targets) and therefore cannot be regulated like its larger counterparts. However, in contrast to these proteins, expression of Bim_S_ appears to be highly restricted *in vivo*, and its precise role remains unclear [20], [21].

Here we identify a novel mechanism of Bim inactivation, via nuclear sequestration, mediated by HHV-8 vIRF-1. The data presented suggest that disruption of such regulation could provide a unique and useful means of controlling HHV-8 productive replication.

## Results

### Identification of nuclear-localized Bim

**Figure 1:**
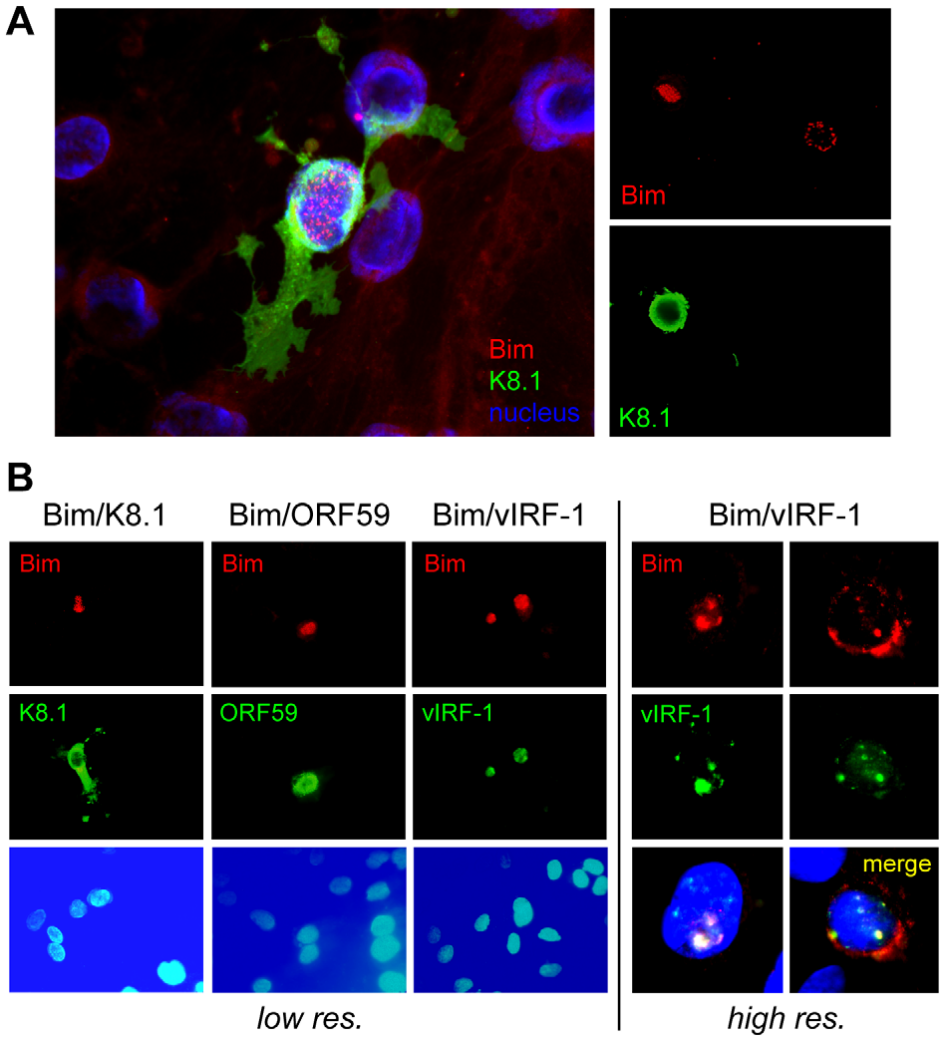
Nuclear localization of Bim during HHV-8 productive replication. (A) Telomerase-immortalized endothelial (TIME) cells latently infected with HHV-8 were treated with TPA to induce lytic replication. Cells supporting lytic reactivation were identified by immunofluorescence staining for K8.1 late lytic membrane protein and Bim was detected by co-staining with appropriate immunological reagents (see Materials and Methods), six days after lytic induction. The left panel shows merged immunofluorescence staining for K8.1 (green) and Bim (red) together with Hoechst nuclear staining (blue). The right panels show individual staining for K8.1 and Bim, emphasizing the correlation of Bim nuclear staining with lytic antigen (K8.1) expression. (B) Analogous confocal immunofluorescence analyses verified Bim nuclear localization specifically in lytically reactivated cells, expressing ORF59-encoded and vIRF-1 early nuclear antigens in addition to K8.1 late membrane protein, and revealed colocalization of vIRF-1 and Bim staining patterns.

Whilst we previously observed that HHV-8 encoded chemokine signaling led to diminished Bim expression [12], immunofluorescence analysis of Bim expression in HHV-8 lytically infected endothelial cells revealed that detectable Bim was largely sequestered in the nuclei, rather than in the cytoplasm where it is normally localized and known to function. Thus, co-staining for Bim and K8.1 late lytic antigen, to identify cells supporting lytic reactivation in HHV-8^+^ telomerase-immortalized endothelial (TIME) cells [22], enabled correlation of lytic infection with Bim nuclear localization (Fig. 1A). Co-staining for early (vIRF-1, ORF59) lytic antigens, in addition to K8.1, again demonstrated Bim nuclear localization specifically in cells supporting lytic reactivation, providing verification of this phenomenon (Fig. 1B). Also revealed in this experiment was correspondence of nuclear staining patterns of Bim and vIRF-1, suggesting the possible involvement of vIRF-1 in Bim nuclear localization. It is worth noting that while some cytoplasmic Bim staining was occasionally detected in cells co-staining for lytic antigen, nuclear Bim staining was always predominant, and nuclear-localized Bim was never detected in mock-infected cultures (+TPA).

### vIRF-1:Bim interactions

**Figure 2:**
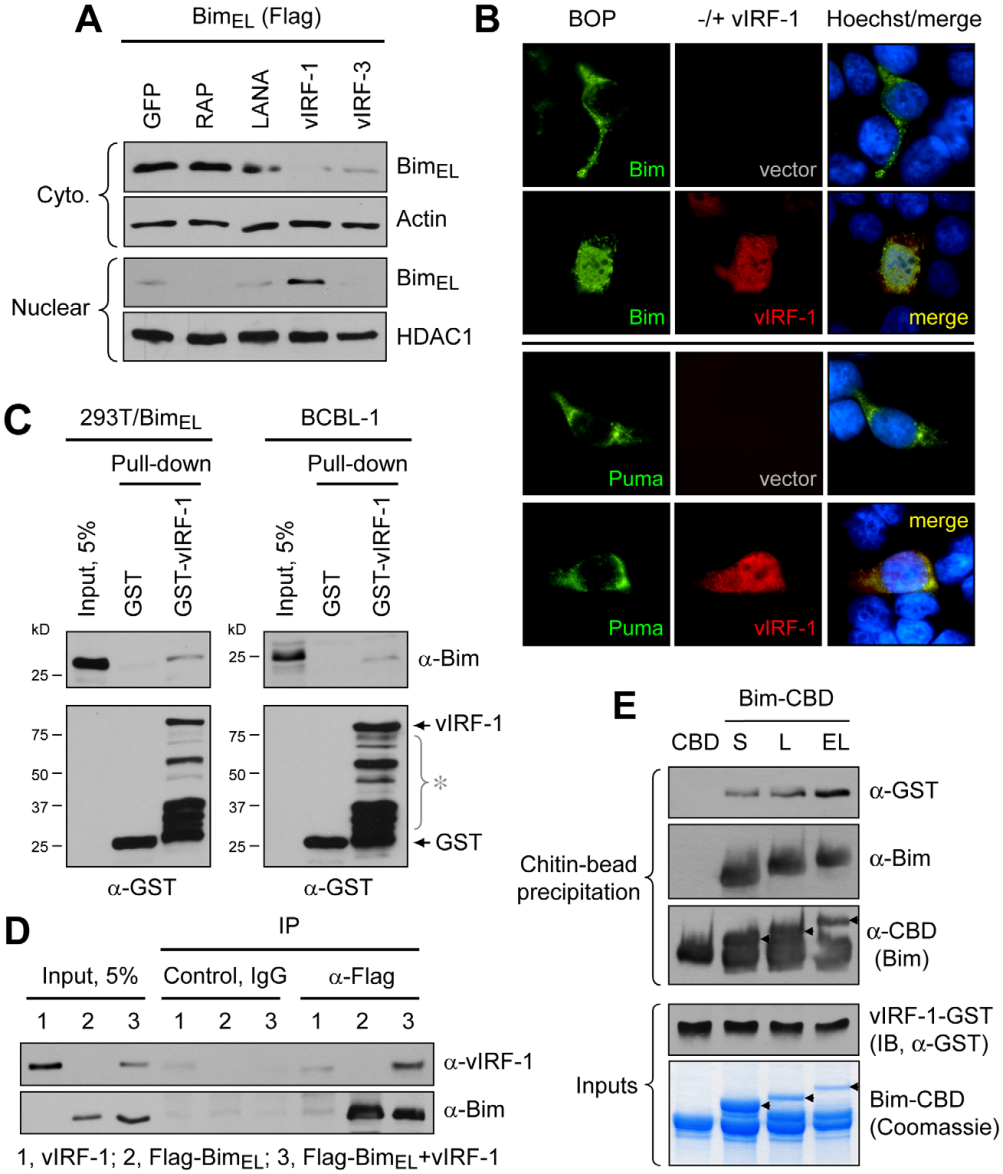
vIRF-1 induced Bim nuclear localization and vIRF-1:Bim interaction. (A) vIRF-1 and selected other HHV-8 nuclear proteins, or GFP (negative control), were tested for their abilities to induce nuclear translocation of Bim_EL_-Flag in transfected HEK293T cells by western analysis of cytoplasmic and nuclear fractions of transfected cells. vIRF-1, specifically, induced nuclear localization of Bim. (B) IFA-determined nuclear translocation of Flag-Bim_EL_ (but not Flag-Puma, control BH3-only protein) induced by vIRF-1 in expression vector-transfected HEK293T cells. Nuclear localization of Bim in vIRF-1 co-transfected cells correlated with strong nuclear vIRF-1 staining. (C) Physical association of vIRF-1 and Bim was detected by co-precipitation assay employing GST-fused vIRF-1 mixed with extracts of Flag-Bim_EL_ vector-transfected HEK293T or untransfected BCBL-1 cells [24], the latter expressing high levels of endogenous Bim. Western analysis of glutathione bead-precipitated material identified Bim precipitated with GST-vIRF-1 protein, but not with GST alone (top panels). Lower panels show precipitated GST protein; bracketed bands (*) correspond to GST-vIRF-1 protein degradation products. (D) Association of Bim and vIRF-1 in intact cells as evidenced by vIRF-1 co-precipitation with Flag antibody-immunoprecipitated (IP) Flag-Bim_EL_ from cell lysate of appropriately transfected cells. Cntl IgG, control (non-specific) IgG. (E) *In vitro* binding assay using bacterially expressed and purified recombinant GST-vIRF-1 and chitin-binding domain (CBD)-fused Bim_S_, Bim_L_ and Bim_EL_ isoforms, precipitable with chitin beads. All Bim-CBD constructions, but not CBD alone, were able to co-precipitate GST-vIRF-1, demonstrating direct binding of each Bim isoform to vIRF-1.

To investigate the potential role of vIRF-1 in mediating nuclear localization of Bim, cotransfection assays (in HEK293T cells) were employed. Expression vectors for Bim_EL_ [extra-large isoform [23], Flag-tagged] and vIRF-1, in addition to other HHV-8 nuclear proteins or GFP negative control, were utilized in these experiments. Cytoplasmic versus nuclear distribution of Bim in the absence and presence of the co-expressed viral proteins was determined by immunoblotting of the respective fractions. The results showed that vIRF-1, specifically, induced nuclear translocation of Bim (Fig. 2A). This was verified in intact cells by immunofluorescence assay (IFA); vIRF-1, co-expressed with Bim_EL_ in transfected cells, was able to induce nuclear translocation of the BH3-only protein (Fig. 2B, top), consistent with the western data. This effect was not seen with Puma, another pan-Bcl-2-binding BH3-only protein, demonstrating specificity of the effects seen with Bim (Fig. 2B, bottom). Whether vIRF-1 was able to interact (directly or indirectly) with Bim was tested by utilizing glutathione-S-transferase (GST)-fused bacterially-derived recombinant vIRF-1 in co-precipitation assays. GST-vIRF-1 was added to lysates of Flag-Bim_EL_ transfected HEK293T cells or to lysates of BCBL-1 (PEL) cells [24], naturally expressing high levels of Bim, and glutathione bead-precipitated material analyzed by immunoblotting. Bim was co-precipitated in a vIRF-1-dependent manner (Fig. 2C). Evidence of vIRF-1:Bim interaction was obtained also from immunoprecipitations from lysates of cells cotransfected with Flag-Bim_EL_ and vIRF-1 expression vectors (Fig. 2D). Direct interaction between vIRF-1 and Bim was demonstrated by co-precipitation assays utilizing bacterially-expressed and purified proteins, fused to GST and chitin-binding domain (CBD) sequences, respectively. vIRF-1 could be co-precipitated with CBD-fused Bim (short, long and extra-long isoforms), but not with CBD alone, following sedimentation with chitin beads (Fig. 2E). Together, these data provided evidence of vIRF-1:Bim association and of vIRF-1-mediated Bim nuclear translocation. That vIRF-1:Bim co-localization was seen in cells lytically infected with HHV-8 (Fig. 1B) suggested biological relevance of vIRF-1:Bim interaction.

### Mapping of Bim-interacting region of vIRF-1

**Figure 3:**
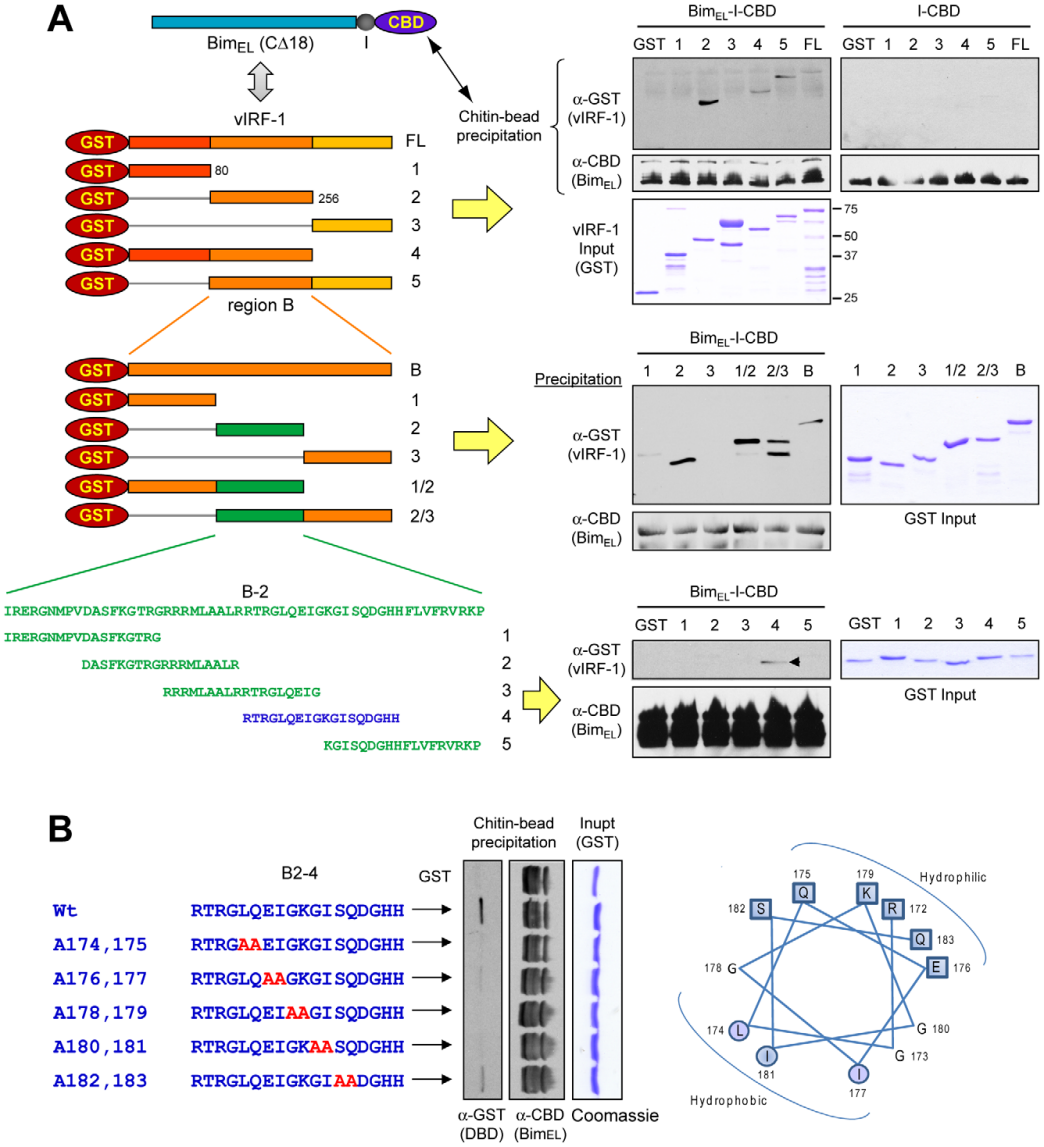
Mapping Bim interaction site of vIRF-1. (A) Recombinant GST-vIRF-1 (progressively deleted) and Bim_EL_-CBD proteins were used in co-precipitation-based binding experiments to determine the “Bim-binding domain” (BBD) of vIRF-1. The C-terminal hydrophobic region of Bim_EL_ (last 18 amino acids) was deleted to enhance solubility of the protein in bacteria. The binding assay employed was analogous to that of Fig. 2E. (B) Mutations introduced into the deletion-mapped BBD (residues 170–187) identified residues 174–181 as necessary for interaction with Bim.

As vIRF-1 is known to interact with several cellular proteins, such as IRFs, p53, ATM, GRIM19 and Smads [25], [9]–[11], [26], precise mapping of its interaction with Bim was necessary to enable experimental assessment of the functional significance of vIRF-1:Bim interaction, specifically. To this end, a series of successively refined deletion variants of vIRF-1 were generated as bacterially expressed GST-fusion proteins for use in *in vitro* co-precipitation assays, along with CBD-fused Bim_EL_. Full-length vIRF-1-GST and derivatives containing the central region (residues 80–256) could be co-precipitated with Bim_EL_-CBD using chitin beads, demonstrating involvement of these sequences in binding (Fig. 3A, top). Further deletion analysis revealed that the central portion of this region was sufficient for binding (Fig. 3A, middle). Based on this result, sequences coding for overlapping 18-mer peptides derived from this central portion were cloned to further map the Bim-binding sequences. The region corresponding to residues 170–187 (peptide-4) was sufficient for association with Bim_EL_ in this assay (Fig. 3A, bottom). Mutations within this putative amphipathic α-helical region were introduced to fine-map the Bim-binding region of vIRF-1; mutation of the central residues (174–181) abrogated interaction (Fig. 3B). Therefore, a region of vIRF-1 sufficient for direct interaction with Bim_EL_ was mapped to vIRF-1 residues 170–187, the core amino acids 174–181 of this Bim-binding domain (BBD) being required for binding. This region of vIRF-1 is divergent from collinear regions of other IRFs, both viral and cellular.

### vIRF-1 BBD involvement in Bim nuclear localization

**Figure 4:**
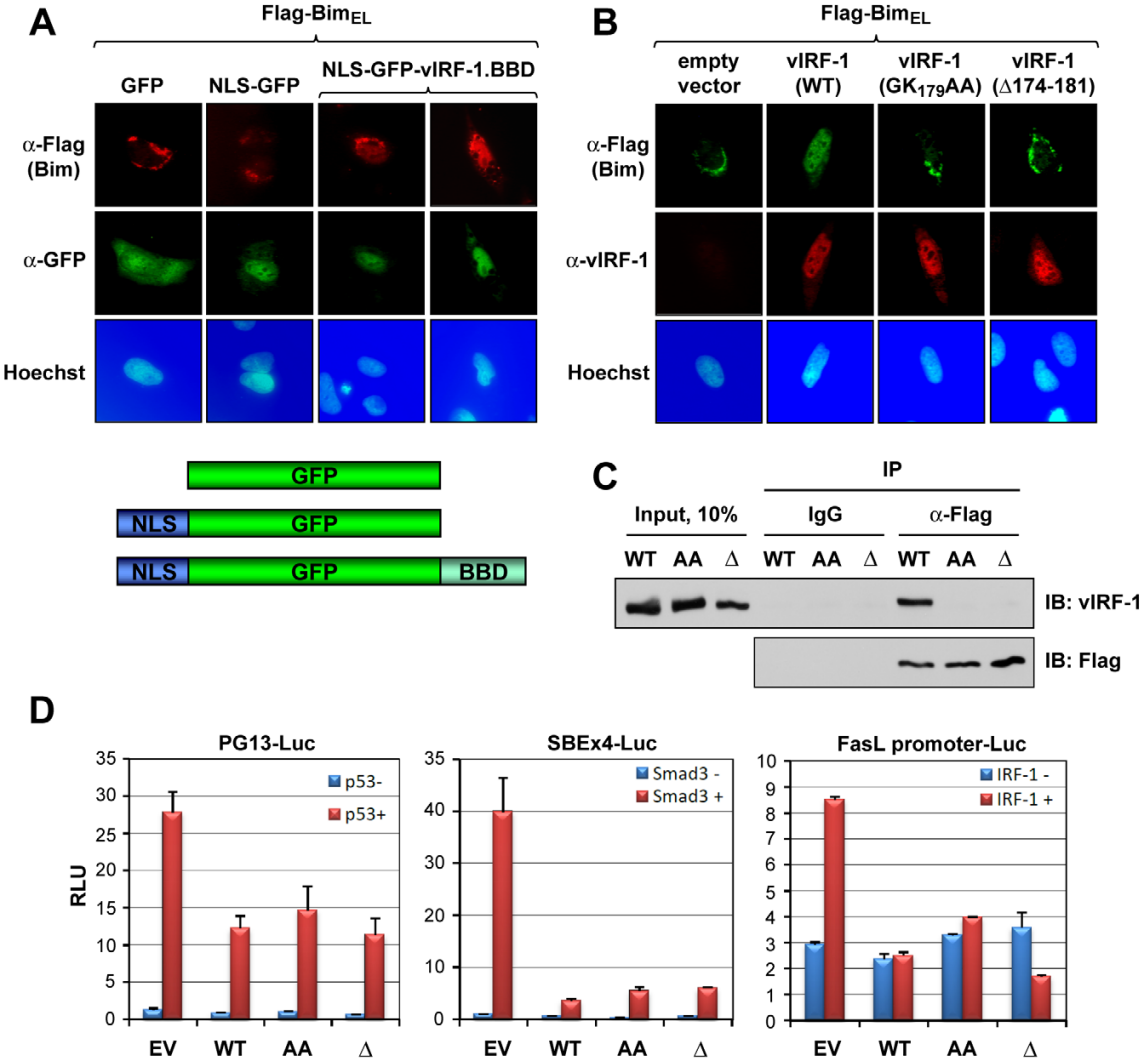
Sufficiency and requirement of vIRF-1 BBD for Bim interaction. (A) Sufficiency of BBD for Bim binding intracellularly was determined by immunofluorescence assay (IFA) in cells transfected with plasmid vectors expressing nuclear localization signal (NLS)-fused BBD (also linked to GFP) and Flag-Bim_EL_. NLS-GFP was used as a negative control. (B) Analogous IFA studies employing BBD core residue-deleted (Δ174-181) or -substituted (GK_179_AA) versions of vIRF-1 verified the importance of BBD in the context of full-length vIRF-1 for interaction with and nuclear localization of Bim. (C) BBD-altered vIRF-1 proteins also were unable to bind Bim_EL_ in immunoprecipitation (IP) binding assays (analogous to those in represented in Fig. 2D). (D) Reporter assays were used to assess the specificity of effects of BBD alteration, as BBD lies within (large) regions of vIRF-1 previously shown to interact in inhibitory fashion with p53, Smads 3 and 4, and IRF-1 [25], [10], [11]. Luciferase reporters responsive to these proteins were inhibited equivalently by wild-type and BBD-mutated vIRF-1 proteins, in the presence of p53, Smad3 or IRF-1. Results are from three transfections; error bars represent standard deviations from means values.

Next, the relevance of the BBD region to vIRF-1-mediated nuclear localization of Bim was examined. First, the 170–187 BBD of vIRF-1 was tested for its ability to bind to and effect nuclear localization of Bim when linked to a nuclear localization signal (NLS). Sequences encoding BBD and NLS were fused to the GFP open reading frame in a eukaryotic expression vector; a vector specifying GFP-NLS, lacking the 18-mer BBD coding sequence, was made to provide a control. The former, specifically, was able to induce nuclear translocation of Bim_EL_ in appropriately transfected cells, as determined by IFA (Fig. 4A), demonstrating sufficiency of the mapped sequences for interacting with Bim_EL_ intracellularly and enabling its nuclear translocation (directed by NLS). The requirement of the 174–181 region and core residues 178/179 of vIRF-1 BBD for Bim nuclear translocation in the context of full-length vIRF-1 was demonstrated in analogous experiments utilizing wild-type and BBD core-deleted or -mutated vIRF-1 (Fig. 4B). The requirement of these residues for Bim interaction was determined directly by co-precipitation assay using lysates of HEK293T cells transfected with expression vectors for wild-type or BBD-mutated/deleted vIRF-1 proteins and Flag-tagged Bim_EL_ (Fig. 4C). Although disrupting Bim interaction and nuclear localization by vIRF-1, the substitution and deletion mutations had no significant effects on functional interactions of vIRF-1 with p53, Smad3 and IRF-1 having potentially overlapping interactions with the central region of vIRF-1 [25], [27], [10], [11] (Fig. 4D). Thus, the introduced mutations disrupted vIRF-1 interaction with Bim specifically.

### Apoptotic regulation via vIRF-1:Bim interaction

**Figure 5:**
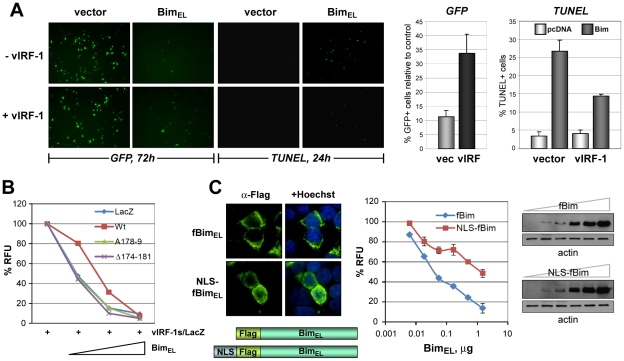
Functional significance of vIRF-1:Bim binding and Bim nuclear translocation. (A) vIRF-1 inhibition of Bim-induced cell death and apoptosis was identified using GFP-based cell viability and TUNEL assays, respectively. The left panels show examples of fluorescence microscopy results from which quantified data (right panels, charts) were derived. (B) Using the GFP-based assay and quantifying GFP fluorescence by fluorometry, Bim-inhibitory activity of wild-type vIRF-1 was compared against activities of BBD-altered vIRF-1(GK_179_AA) and vIRF-1Δ174-181. The latter were unable to inhibit Bim_EL_ activity. (C) Using the same assay, the functional consequence of nuclear translocation of Bim was tested using an NLS-fused version of Bim_EL_ (Flag-tagged, “fBim”). NLS-fBim_EL_, localizing predominantly in the nuclei of transfected cells as expected (IFA, bottom panels), was significantly inhibited relative to native fBim_EL_ in respect of pro-death activity (chart, middle panel). Results are expressed in relation to GFP fluorescence values (100%) derived from control cultures transfected with empty vector instead of Bim vectors. Western blotting (right panels) verified equivalent expression of native and NLS-fused versions of Flag-tagged Bim_EL_; fBim- and NLS-fBim-containing extracts were processed identically and in parallel, and therefore band intensities are directly comparable. Data from apoptosis assays were derived from triplicate (panels A and B) or duplicate (panel C) transfections; error bars show standard deviations from mean values. GFP fluorescence measurements were undertaken 24 h posttransfection (panels B and C).

The functional consequence of vIRF-1 expression on cell viability and apoptosis in response to Bim_EL_ was examined using GFP- and TUNEL-based assays. In the former, GFP expression and fluorescence was diminished as a function of Bim_EL_ plasmid transfection and therefore the proportion of GFP^+^ cells in the population, determined by counting of cells under UV microscopy (and using co-staining with Hoechst to visualize nuclei), provided a measure of cell viability. Both apoptosis (identified by TUNEL staining) and cell viability were inhibited significantly (>50% under the conditions used) by vIRF-1 co-expression (Fig. 5A). Using the GFP viability assay and quantifying GFP fluorescence by fluorometry, we found that in contrast to wild-type vIRF-1, the Bim-refractory vIRF-1 deletion and point variants, vIRF-1(Δ174-181) and vIRF-1(GK_179_AA), were unable to inhibit of Bim_EL_-induced cell death (Fig. 5B). That nuclear translocation of Bim induced by vIRF-1 could theoretically account for the observed decrease in Bim_EL_-induced apoptosis was verified utilizing an NLS-fused version of Bim_EL_, which accumulated predominantly in the nuclei of transfected cells (Fig. 5C, left). This construction was greatly impaired relative to native Bim_EL_ with respect to apoptotic induction, although expressed equivalently (Fig. 5C, middle and right panels), confirming the inhibitory effect of nuclear sequestration of the normally cytoplasmic protein. Combined, these data suggest that pro-apoptotic functions of Bim are lost upon nuclear localization and that inhibition of Bim activity by vIRF-1 is mediated via direct interaction that enables cytoplasmic-to-nuclear translocation of the BH3-only protein by vIRF-1.

### Relevance of vIRF-1:Bim interaction to virus replication

**Figure 6:**
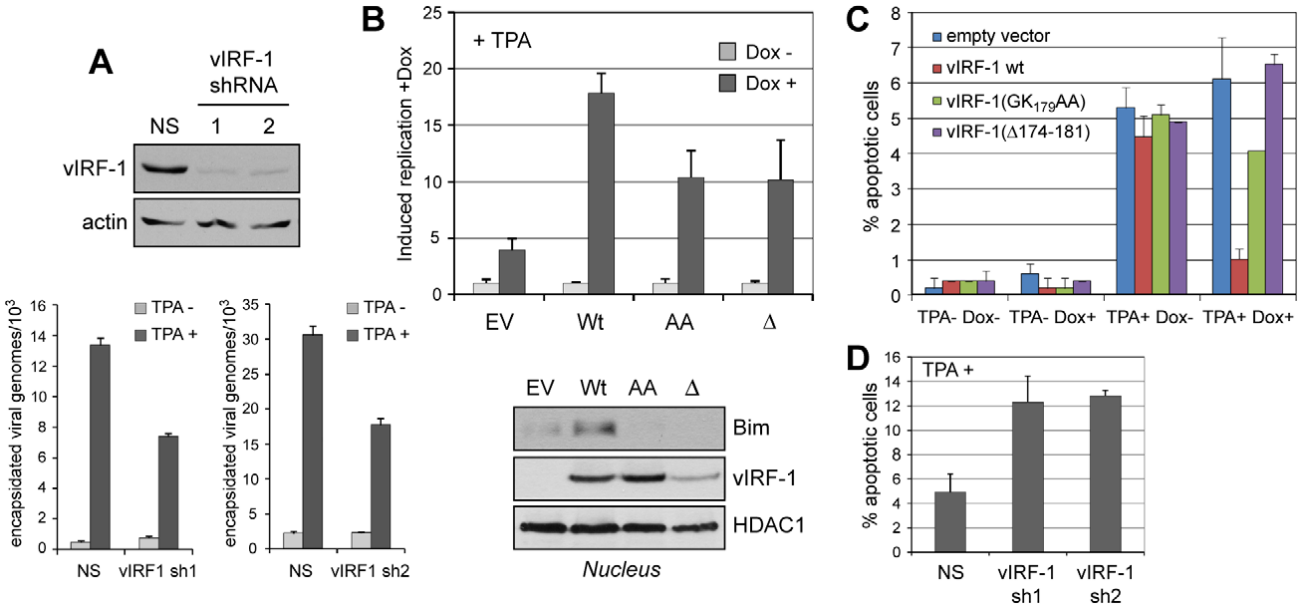
vIRF-1 function and significance of vIRF-1:Bim interaction in the context of virus infection. (A) Two lentiviral-cloned shRNAs effective for vIRF-1 depletion (top panels) were transduced into HHV-8 (latently) infected TIME cells and lytic reactivation then induced with TPA. After six days, encapsidated (DNaseI-resistant) viral genomes released into culture media were quantified by qPCR [12]. vIRF-1 shRNA-specific reductions of viral titers were observed. NS, non-silencing shRNA control. Data were obtained from triplicate qPCR reactions; error bars show standard deviations from mean values. (B) Effects of wild-type and BBD-altered [GK_197_AA (“AA”) and Δ171-184 (“Δ”)] vIRF-1 proteins on virus replication were tested using TIME cell lines expressing each of the proteins in a doxycycline (Dox)-inducible fashion. EV, empty vector control. Data were derived from triplicate PCR reactions; error bars show deviations from mean values. Western analysis of nuclear extracts revealed induced nuclear localization of endogenous Bim only in wild-type vIRF-1 expressing cells. (C) In analogous experiments, TUNEL analysis of apoptosis revealed effective protection from lytic cycle-induced apoptosis (+TPA) by overexpression of wild-type vIRF-1 (+Dox), but not by Dox-induced vIRF-1 variants GK_179_AA or Δ174-181. Data were derived from multiple random fields for each condition; error bars represent standard deviations from mean values. (D) Similar analysis of apoptosis in vIRF-1-depleted and control non-silencing (NS) shRNA-transduced TIME cultures revealed significantly increased rates of HHV-8 replication-induced apoptosis in vIRF-1 shRNA (sh1, sh2) expressing cultures, demonstrating anti-apoptotic function of endogenously expressed vIRF-1. Data were derived multiple random fields from duplicate cultures; error bars reflect deviations from mean values obtained from each.

To address biological significance, vIRF-1 function and vIRF-1:Bim interaction in the context of HHV-8 lytic replication were examined. First, lentiviral vectors were generated specifying vIRF-1-targeted shRNAs for vIRF-1 depletion, or non-silencing (NS) control shRNA, to determine whether vIRF-1 contributed detectably to virus productive replication in culture. TPA-induced virus production from HHV-8 latently infected telomerase immortalized endothelial (TIME) cells was markedly reduced in vIRF-1-depleted relative to control (NS shRNA-transduced) cultures, as determined by qPCR applied to DNaseI-pretreated and -resistant (encapsidated) viral DNA (Fig. 6A).

Having identified pro-replication activity of endogenously produced vIRF-1, we next generated TIME cell cultures expressing Dox-inducible vIRF-1, vIRF-1(Δ174-181) or vIRF-1(GK_179_AA) to assess the relative abilities of the wild-type and Bim-refractory vIRF-1 proteins to enhance HHV-8 productive replication. While all proteins enhanced virus production in Dox-treated cells, the Bim-refractory vIRF-1 variants were significantly less active, demonstrating the contribution and importance of Bim interaction, specifically, to pro-replication activity (Fig. 6B, top). Western analysis of Bim nuclear localization in these cultures revealed vIRF-1-enhanced nuclear localization of Bim, relative to empty vector (EV) control, and apparent reductions of nuclear Bim in cultures expressing the BBD variants of vIRF-1. The latter suggests possible dominant negative activity of these Bim-refractory proteins, although the mechanism that might be involved is not clear. Nonetheless, these data demonstrate that vIRF-1 is able to induce nuclear localization of Bim in the context of virus productive replication. In parallel experiments, apoptosis induced in infected cells (positive for latency-associated nuclear antigen, LANA) upon TPA treatment was substantially reduced in vIRF-1 overexpressing cultures (+Dox), but the BBD-mutated vIRF-1 proteins displayed little or no activity (Fig. 6C). These data implicate vIRF-1:Bim interactions as centrally important for vIRF-1-mediated protection from lytic cycle-induced apoptosis. In shRNA-depleted cultures, rates of apoptosis upon TPA treatment of HHV-8 infected TIME cultures were induced ∼2.5-fold relative to control NS shRNA-transduced cultures, revealing the significant contribution of endogenously expressed vIRF-1 to suppression of lytic cycle-induced apoptosis (Fig. 6D). Together, these data indicate that vIRF-1 and vIRF-1:Bim interaction, specifically, are effective mediators of apoptotic inhibition during lytic replication and, in combination with the replication experiments (Fig. 6B), that this is important for establishing conditions conducive to efficient virus production.

### Inhibition of vIRF-1:Bim interaction

**Figure 7:**
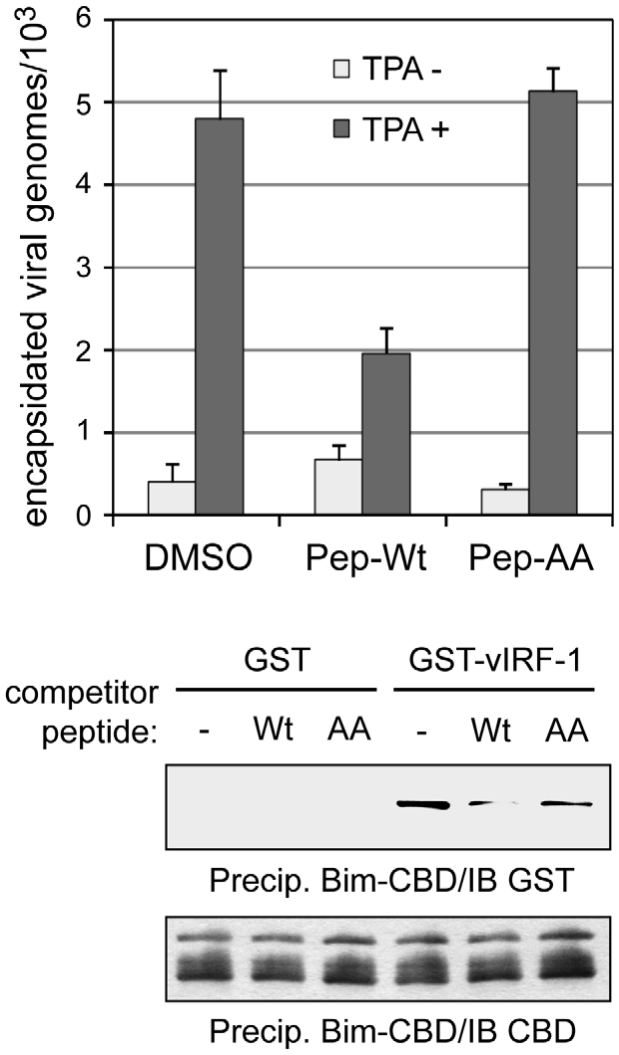
BBD peptide-mediated disruption of vIRF-1:Bim interaction. The biological significance of Bim binding by vIRF-1 and feasibility of targeted disruption of vIRF-1:Bim interaction were tested using cell-permeable (Tat-fused) BBD peptides, wild-type and AA-mutated. These were applied (at 10 µM final concentration) to HHV-8 infected TIME cultures prior to TPA induction. Inhibition of virus production specifically by wild-type BBD (top) reflected the relative abilities of the wild-type and mutated peptides to interfere with vIRF-1:Bim interaction *in vitro* (lower panels). Replication data were derived from triplicate PCR reactions; error bars indicate deviations from mean values.

The role of vIRF-1:Bim interaction, specifically, independent of vIRF-1 interactions with other cellular proteins, was further demonstrated by addition in replication experiments of Tat-fused (cell-permeable) peptides corresponding to Bim-interacting vIRF-1 residues 170–187 or Bim-refractory GK_179_AA-mutated equivalent. The wild-type peptide, specifically, led to reduced virus titers (Fig. 7). Again, these data indicate that vIRF-1:Bim interaction and inhibition of Bim pro-apoptotic activity are important for virus productive replication. The results further suggest that disruption of vIRF-1:Bim, perhaps through the use of small molecule inhibitors, could potentially provide a means to inhibit virus replication specifically and for therapeutic benefit.

## Discussion

HHV-8 encoded viral interferon regulatory factors have been noted previously to interfere with innate immune responses of cells, in particular via their inhibition of activities of cellular IRFs, necessary for interferon induction and consequent cell cycle arrest and apoptosis [6], [8]. vIRF-1 has been reported to interact with a broad range of proteins in addition to cellular IRFs; these other proteins include p53, ATM, TGFβ-activated Smad transcription factors, and GRIM19 [25], [9], [10], [26], all of which play roles in mediating innate immune functions via regulation of cell cycle and apoptosis. These activities, induced by infection, presumably must be controlled sufficiently by the virus to allow productive replication in the face of stress signaling. Bim, induced strongly and rapidly following HHV-8 lytic reactivation in latently infected endothelial cells, is a powerful inhibitor of HHV-8 production in this cell type and thus represents a biologically relevant target of vIRF-1 [12]. The data presented here indicate that vIRF-1:Bim interaction is indeed important in the context of virus replication, being necessary for substantial or major proportions of replication-enhancing and anti-apoptotic activities specified by vIRF-1 in lytically reactivated endothelial cells. Thus, vIRF-1 regulation of Bim pro-apoptotic function represents a critical component of vIRF-1 activity and one which is essential for normal virus productive replication, at least in this cell type.

To our knowledge, this is the first demonstration of interaction between an IRF homologue and a member of the Bcl-2 family, and the first report of nuclear translocation of Bim or any other Bcl-2 related protein as a means of functional inactivation. Whether physiological conditions unconnected with viral infection can promote such nuclear localization and inactivation is unclear; none has been reported to date. On the other hand, several previous investigations have noted the nuclear localization and functions of Bcl-2 family members. Bcl-2 nuclear localization can occur during oxidative stress, Bcl-2 overexpression or loss of interaction with mitochondrial-localizing protein FKBP38, with promotion of apoptosis via blocking of nuclear trafficking of transcription factors [28]–[31]. Mcl-1 has been reported to inhibit cell proliferation via inhibitory interactions with proliferating cell nuclear antigen and cyclin-dependent kinase 1 [32]–[34], whereas the Bcl-2-related protein Bok, which cannot heterodimerize with Bcl-2 or Bcl-x_L_, can localize to and function in the nucleus to promote apoptosis [35]. A splice variant of Bfl-1, Bfl-1S, in which mitochondrial-localizing hydrophobic C-terminal sequences are replaced with a basic nuclear-localization signal, may mediate anti-apoptotic activity via nuclear sequestration of components of the apoptotic cascade [36]. Nuclear-localized pro-death activity of apoptotic effector Bax has been proposed due to correlation of Bax nuclear translocation in response to alkylating agent (BCNU) with glioma cell sensitivity to the apoptotic inducer [37]. Thus, while there is prior evidence of nuclear localization of Bcl-2 family proteins, both pro- and anti-apoptotic, along with evidence of function in this compartment, the induced nuclear localization and inactivation of BH3-only protein Bim identified here appears to be unique and the first example of viral control of apoptosis via nuclear sequestration of a Bcl-2 family member. That a viral IRF homologue mediates this effect is also a novel finding. Our demonstration that vIRF-1:Bim interaction is both important for virus productive replication and can be inhibited via peptide-mediated disruption suggests that targeting vIRF-1:Bim interaction may provide a useful antiviral strategy.

## Materials and Methods

### Plasmids and expression vectors

Two short hairpin RNAs (shRNA) for vIRF-1 were cloned into pYNC352/puro (a derivative of pYTF [38]) using *Bam*HI and *Mlu*I enzyme sites; target sequence of the shRNAs correspond to 5′- AGCCGGACACGACAACTAAGA -3′ (sh1) and 5′-ATCAAGGATTGGATAGTATGT-3′ (sh2). Sequences specifying wild-type or mutated forms of vIRF-1 were cloned into lentivirus vector pYNC352/SV40/puro using *Mlu*I and *Bam*HI cloning sites. Bim_EL_ cDNA sequences linked to Flag were cloned between the *Bam*HI and *Eco*RI sites of pcDNA3.1 (Invitrogen; Carlesbad, CA), for expression in transfected cells. Coding sequence for the nuclear localization signal (NLS) of SV40 large T antigen was inserted between the *Hind*III and *Bam*HI sites of pcDNA3.1-flag-Bim_EL_ to generate a eukaryotic expression vector encoding NLS-flag-Bim_EL_. Bacterial expression plasmids for Bim_EL_ and vIRF-1 were generated by cloning of the respective coding sequences into pTYB4 (New England Biolabs; Ipswich, MA) and pGEX-4T-1 (GE Life Sciences; Piscataway, NJ), using *Nco*I and *Sma*I sites and *Bam*HI and *Eco*RI sites, respectively. The Bim_EL_ and vIRF-1 proteins were fused to intein/chitin-binding-domain (CBD) and GST, respectively, used for precipitation and purification via chitin- and glutathione-bead capture. Fas ligand promoter sequences encompassing 1.2-kb upstream of the initiator codon [39] were amplified from BCBL-1 cell DNA by PCR and cloned between the *Xho*I and *Hind*III sites of pGL3/basic to provide a reporter construction responsive to IRF-1. The NLS and BBD coding sequences were cloned between *Kpn*I and *Age*I and *Bsr*GI and *Xba*I sites, respectively, of pEGFP-N1 (Clontech Laboratories; Mountainview, CA) to generate nuclear-directed GFP and GFP-BBD proteins. Coding sequences for wild-type or mutated vIRF-1 proteins were cloned between the *Bam*HI and *Mlu*I sites of pRetroX-Tight-Pur (Clontech Laboratories) to construct viral vectors for the generation of TIME cultures conditionally expressing the proteins (+Dox).

### Cell culture, transfection and viral transduction

TIME cells [22] were maintained in EGM-2 MV medium (Lonza, Walkersville, MD) containing 5% fetal bovine serum (FBS) and cytokine supplements. HEK293 and HEK293T cells were grown in Dulbecco's modified Eagle's medium supplemented with 10% FBS and gentamicin. BCBL-1 cells [24] were cultured in RPMI 1640 supplemented with 20% heat-inactivated FBS and gentamicin. For lentivirus production, HEK293T cells were transiently transfected with virus vector and gag/pol-encoding packaging plasmids using standard calcium-phosphate precipitation method and virus harvested after 48 h by centrifugation at 49,000×g, essentially as described previously [12]. Other transfections were performed using Lipofectamine 2000 (Invitrogen). Stable transduction of shRNA or cDNA into TIME cells using lentivirus vectors was performed under puromycin selection.

### Cell extracts, immunoblotting and immunofluorescence

For whole cell extracts, cells were lysed in lysis buffer (50 mM Tris-HCl [pH 8.0], 150 mM NaCl, 1 mM EDTA, 1% IGEPAL CA-630, 0.25% sodium deoxycholate, and protease inhibitor cocktail). For nucleo-cytoplasmic fractionation, cells were homogenized in buffer A (10 mM HEPES [pH 8.0], 1.5 mM MgCl_2_, 10 mM KCl, 0.5 mM DTT, and protease inhibitor) using a Dounce homogenizer. After centrifugation of the homogenate at 1,500×g, the supernatant was used as the cytoplasmic fraction and the pellet, after resuspended in buffer B (20 mM HEPES [pH 8.0], 1.5 mM MgCl_2_, 420 mM NaCl, and 0.2 mM EDTA), was used as the nuclear fraction. For immunoblotting, proteins were size fractionated by sodium dodecyl sulfate-polyacrylamide gel electrophoresis (SDS-PAGE) and transferred to a nitrocellulose membrane. Immunoreactive bands were detected with enhanced chemiluminescence solution (GE Healthcare, Piscataway, NJ) and visualized on X-ray film. For immunofluorescence assay (IFA), cells on a 0.1% gelatin-coated coverglass were fixed and permeabilized in chilled methanol. Following incubation with superblock blocking buffer (Thermo Scientific Inc., Rockford, IL), coverslips were incubated with primary antibody, washed with phosphate-buffered saline (PBS), and then incubated with appropriate fluorescent dye-conjugated secondary antibody. Coverslips were mounted in 90% glycerol in PBS containing 10 mg/ml *p*-phenylenediamine. Nuclei were visualized by staining with Hoechst 33342.

### HHV-8 infection, replication, and quantitative PCR

Infectious HHV-8 was obtained by inducing BCBL-1 cells with phorbol 12-myristate 13-acetate (PMA/TPA; 20 ng/ml) and calcium ionophore (A23187; 500 ng/ml). After 20 h, cells were pelleted and resuspended in fresh medium without TPA and A23187. After four days, virions were pelleted from culture media by centrifugation at 27,000×g for 2 h in an SW41 rotor and resuspended in basal EGM-2 MV medium. For HHV-8 infection, TIME cells were centrifuged at 1,000×g for 1 h in the presence of HHV-8 virions, and then cultured in fresh complete medium for 7 days to allow establishment of latency in the absence of ongoing lytic replication. Lytic replication of HHV-8 in TIME cells was induced by treatment with TPA. For determination of encapsidated HHV-8 genome copy number, viral DNA was extracted using guanidinium thiocyanate (6M) and silica gel following pre-treatment of virus suspensions with DNaseI for 20 min. at 37°C. Excess HHV-8 bacmid DNA was treated with DNaseI and processed identically to control for DNaseI efficacy. All qPCRs were performed in a 96-well microplate using an ABI Prism 7500 detection system (Applied Biosystems; Foster City, CA) with SYBR green/ROX master mix (SuperArray Bioscience Corp.; Frederick, MD). Reactions were performed in a total volume of 25 µl, containing viral DNA sample and 250 nM of each primer. To calculate copy number of viral DNA, BAC-cloned HHV-8 genomic DNA was used as a standard. PCR conditions included an initial incubation step of 2 min. at 50°C, and enzyme heat activation step of 10 min at 95°C, followed by 45 cycles of 15 seconds at 95°C for denaturing and 1 min at 60°C for annealing and extension.

### Reporter assay

HEK293T cells were transiently transfected with plasmids expressing vIRF-1 and p53, Smad3 or IRF-1 along with reporter plasmids, PG13-luc (Addgene; Cambridge, MA), SBEx4-luc (Addgene), or Fas ligand promoter-luc (see above), respectively, for 24 h and then lysed with passive lysis buffer (Promega, Madison, WI). Luciferase activity was measured by standard methods using D-luciferin and luminometry.

### Cell viability assay

Bim-induced cell death of HEK293 cells was monitored by cotransfection of pEGFP-N1 (Clontech laboratories, Mountain view, CA) and measuring fluorescence by fluorometry. For terminal deoxynucleotidyltransferase (TdT)-mediated dUTP-biotin nick end labeling (TUNEL), cells were fixed in chilled methanol for 5 min and preincubated in TdT reaction buffer (25 mM Tris-HCl [pH 6.6], 200 mM sodium cacodylate, 0.25 mg/ml BSA, and 1 mM CoCl_2_) for 10 min. TUNEL reactions were carried out at 42°C for 2 h in TdT reaction buffer containing TdT and biotin-dUTP (Roche, Indianapolis, IN) and terminated with stop solution (300 mM NaCl and 30 mM sodium citrate). TUNEL-positive cells were visualized by staining with FITC-avidin. LANA IFA was performed after TUNEL reactions, using LANA monoclonal antibody (Advanced Biotechnologies Inc; Columbia, MD) and staining with Cy-3-conjugated secondary antibody.

### Protein co-precipitation assay

Glutathione-S-transferase (GST)-fusion proteins were purified by standard methods. Proteins from Bim_EL_-transfected HEK293T or BCBL-1 cells were incubated with purified GST or GST-vIRF-1 proteins immobilized on glutathione beads. After washing with lysis buffer, the bead-precipitated material was subjected to SDS-PAGE and analyzed by immunoblotting using Bim- or GST-specific antibodies. For binding-site mapping, Bim_EL_ (lacking the C-terminal 18 residues, affecting solubility) was fused to intein-chitin binding domain (CBD) in pTYB4 and purified according to the manufacturer's protocol. GST or a series of GST-vIRF-1 fusions were incubated with the purified Bim protein immobilized on chitin beads. After washing with lysis buffer, bead-associated proteins were size-fractionated by SDS-PAGE and analyzed by immunoblotting using GST- or CBD-specific antibodies. For peptide competition assays, peptides (35-fold molar excess) were pre-incubated with Bim_EL_-intein-CBD for 1 h before addition of GST-vIRF-1. Peptide sequences (the first 11 residues comprising Tat basic region) were: BBD-WT, YGRKKRRQRRRGGGRTRGLQEIGKGISQDGHH; BBD-Mut, YGRKKRRQRRRGGGRTRGLQEIAAGISQDGHH (altered residues underlined). For immunoprecipitation, HEK293T cells transfected with plasmids expressing Flag-Bim_EL_ or vIRF-1 were lysed in lysis buffer and cell extracts were incubated with anti-Flag antibody (M2) and immune-complexes precipitated with protein A/G-agarose. After washing with lysis buffer, immune-complexes were subjected to SDS-PAGE and analyzed by immunoblotting using vIRF-1 antiserum, Bim antibody or biotinylated Flag antibody and secondary, detection reagents comprising HRP-conjugated anti-Ig antibody or sptreptavidin-HRP.

### Reagents

K8.1 and LANA antibodies were purchased from Advanced Biotechnologies Inc (Columbia, MD). Antisera directed to vIRF-1 and ORF59 were provided by Drs. Gary Hayward and Bala Chandran, respectively. Actin and Flag antibodies were purchased from Sigma (St. Louis, MO), HDAC1 and GST antibodies from Santa Cruz Biotechnologies, Inc. (Santa Cruz, CA), Bim antibody from Cell Signaling Technologies, Inc. (Beverly, MA), GFP antibody from Epitomics, Inc. (Burlingame, CA), and CBD antibody from New England Biolabs, Inc. (Ipswich, MA).
